# Bioclimatic gradients and soil property trends from northernmost mainland Norway to the Svalbard archipelago. Does the arctic biome extend into mainland Norway?

**DOI:** 10.1371/journal.pone.0239183

**Published:** 2020-09-17

**Authors:** Gauri Bandekar, Live Semb Vestgarden, Andrew Jenkins, Arvid Odland

**Affiliations:** Department of Natural Sciences and Environmental Health, Faculty of Technology, Natural Sciences and Maritime Sciences, University of South-Eastern Norway, Gullbringvegen, Bø i Telemark, Telemark, Norway; Technical University in Zvolen, SLOVAKIA

## Abstract

The boundary between the boreal and arctic biomes in northwest Europe has been a matter of debate for many years. Some authors consider that the boundary is marked by the northern limit of tree growth in the northernmost Norwegian mainland. In this study we have collected air and soil temperature data from 37 heath stands from northern Finnmark (71°N), the northernmost part of the Norwegian mainland, through Bear Island (74°N) in the Barents sea, to Adventsdalen (78)°N (in Spitsbergen) in Svalbard archipelago. In Finnmark, plots both south and north of the treeline were investigated. Vegetation and soil chemistry analyses were performed on the plots in Finnmark and Svalbard. Significant decreasing south-north trends in air and soil temperatures were observed from Finnmark to Spitsbergen. Soils in Finnmark were acidic and rich in organic matter, while those on Adventsdalen were basic and poor in organic matter. Vegetational analysis identified five communities: three in Finnmark and two on Adventsdalen. The communities in Finnmark had marked mutual similarities but were very different from those on Adventsdalen. No significant ecological differences between heaths south and north of the treeline in Finnmark were observed. Air and soil temperature variables in Finnmark were outside the recognized range for the arctic biome and inconsistent with the presence of permafrost both south and north of the treeline. A major difference between Finnmark and Spitsbergen was amount of soil frost and length of the growing season. Our results suggest that the boreal biome extends all the way to the north coast of mainland Norway; and previously used division of heaths in Finnmark into boreal, alpine and arctic biomes is not justified.

## Introduction

In biogeographical terms, the world is divided into biomes: large-scale, climatically-controlled biotic communities whose characteristics are most strongly expressed in the vegetation [1]. The northernmost of these are the arctic, alpine and boreal biomes. Based on differences in vegetation composition and summer temperatures, the arctic biome has been subdivided into five subzones, A to E [2]. Subzone E is the southernmost vegetation zone dominated by shrubs, herbs and bryophytes, moving up north to higher latitudes the vegetation cover reduces with dominance of usually mosses, lichens and few vascular plant species in subzone A [2]. Comprehensive bioclimatic studies conducted in Alaska, Canada and Northern Russia [3–7] show strong gradients of temperature, precipitation, and soil conditions from subzones E to A. The Norwegian arctic islands that is Bear Island and Adventsdalen on Spitsbergen are included in arctic subzone C.

The transition zone (ecotone) between the boreal and the arctic biomes has been variously termed hemiarctic, hemiboreal, tundra forest, forest tundra, subarctic tundra and southern hypoarctic tundra [4, 8–13], with a similar profusion of definitions, which has caused confusion especially when data are compared between countries [14, 15].

Commonly used definition of the boundary between the boreal and arctic biomes is the position of the arctic treeline, north of which the forest gives way to heaths [16, 17]. In theory, these three biomes should meet where the climatic alpine and arctic treelines meet at the sea level. The northernmost parts of the Norwegian mainland (in Finnmark), are treeless heaths and have therefore been equated with arctic subzone E by some authors [2, 16, 18]. The boundary between woodland and heath being defined as the arctic treeline, a line connecting the northernmost limits of woodland across the various peninsulas of the highly-indented northern coast. However, large areas of heaths also occur south of this line, both in high-altitude and low-altitude locations [19].

Problems associated with the separation between arctic and boreal biomes have particularly been difficult in areas strongly influenced by an oceanic climate [20]. Coastal, treeless heaths occur along most of the northern coast of Norway and extend towards east to Kola Peninsula [12, 21]. Criteria developed to define the arctic biome include characteristics of vegetation [8, 12, 16], temperatures [2, 22], permafrost [23] and position north of treeline [2, 16]. Environmental data associated with the northern limit of forests in Norway have, however rarely been studied, except for air temperatures. Data on characteristic environmental conditions of the transition between boreal and arctic have therefore to be based on studies from North America and Siberia.

In exposed, oceanic areas, however, use of occurrence of trees or forest as the only criterion for delimiting arctic might be an unsatisfactory approach [24], and according to Tuhkanen [25], effects of all non-climatic environmental factors are likely to cause deviations from the potentially climatic treeline in the order of tens of kilometers, possibly 100 km in some extreme cases.

The conditions in Finnmark conspire to make definition of the boreal-arctic boundary difficult. Firstly, the North Atlantic Thermohaline Circulation, which draws warm waters from the region of the Gulf of Mexico up along the west coast of Europe and around the northern coast of Norway into the Barents Sea, allows boreal forest to extend far beyond its normal latitudinal limits. Secondly, the proposed arctic treeline lies close to the northern coast of mainland Norway; between there and the unequivocally arctic Svalbard archipelago there is only sea and a few scattered islands. Thus, an orderly progression of arctic subzones cannot be observed. Thirdly, the northern coast of Finnmark is rugged and windswept, so that exposure and altitude further complicate the bioclimatic delimitations. A previous study of ecological conditions within the northernmost birch forests indicated that their distribution might be limited by topography and availability of growth areas rather than by temperature [26].

Coastal heath communities belonging to the boreal, alpine and arctic biomes can be distinguished in terms of various climatic variables, the pivotal variables being growing season length, growing season temperature, heat sum, summer warmth index, temperatures during the warmest month, winter frost and precipitation [4, 22, 27–29]. Both July air temperatures and summer warmth index have been used extensively in high latitude vegetation studies [2], while Karlsen et al. [30] recommend the use of temperature sum. According to Eurola [31], soil temperatures, are of critical importance, but unfortunately the available data is sparse [29, 32, 33]. Soil temperature cannot be simply inferred from air temperature as vegetation cover, albedo and soil frost all influence the relationship between air and soil temperature [32, 34, 35]

Arctic areas are characterized by continuous permafrost, where permafrost underlies more than 80% of the ground surface [36]. The southern limit of continuous permafrost corresponds closely to areas where mean annual air temperature is lower than -8°C. Discontinuous permafrost, where 30–80% of the ground surface is underlain by permafrost, is associated with the subarctic boreal zone. Its southern limit corresponds closely to the mean annual isotherm of -1°C [37, 38].

Permafrost is soil or sediment that remains at or below 0°C for at least two consecutive years [39]. An upper active layer of ca. 50 cm may melt during the summer, leaving the area waterlogged. These conditions are not compatible with the growth of trees. Mean annual soil temperatures measured 20 cm below surface rarely increase above 0°C, but summer temperatures may reach 5°C. Permafrost also influences soil chemistry; leaching is prevented and chemical weathering is retarded, resulting in the retention of cations and high alkalinity, while soil organic carbon levels tend to be low. Boreal soils, on the other hand, are characteristically acid podzols [40].

In this study, we have collected data on vegetation, air temperature, soil temperature and soil properties in order to quantify latitudinal and altitudinal differences in heath vegetation from northernmost mainland Norway, Bear Island and Spitsbergen. These findings extend existing knowledge on coastal heaths on mainland Norway, and we use them to address the question of whether inclusion of the northernmost parts of mainland Norway in the arctic biome is justified.

## Materials and methods

### Study areas

**Fig 1:**
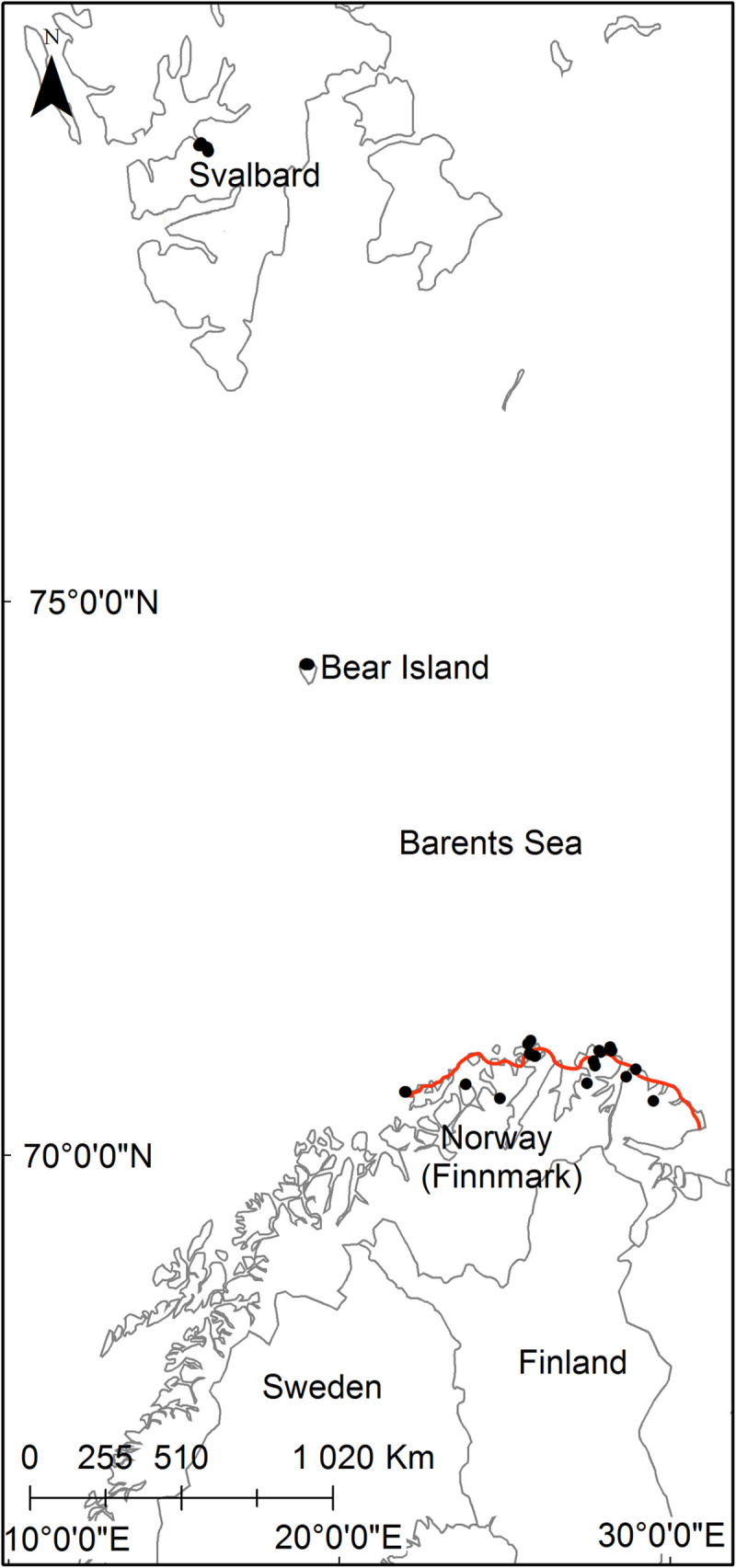
Map of study area. Black points indicate study areas. Red line in figure shows the northernmost distribution limit of birch forests in Finnmark.

Studies were conducted in coastal areas in the northernmost county of Troms and Finnmark on mainland Norway (70–71°N, 21–29°E), Bear Island in the Barents sea (74°N, 18–19°E) and Adventsdalen in Spitsbergen in Svalbard archipelago (78°N, 15–16°E) (Fig 1). Altogether, 37 plots were established at low (< 200 m a.s.l.) and high (> 200 m a.s.l.) elevations; along latitudinal gradient representing several bioclimatic zones.

In Finnmark, the landscape is rugged (although elevation is modest) and the coastline is deeply indented. Much of the coastal region of Finnmark is open to the winds of the Barents Sea with maritime climate, although the rugged topography also affords sheltered areas. The bedrock in Finnmark comprises of sandstone, shale, metamorphic and igneous rocks (Bedrock map of Norway, 1: 1 million, NGU 1984). The main soil types are podzols or shallow leptosols [36]. The proposed position of the arctic treeline described by Elvebakk et al. [16] and Moen [18] was used as a reference line and is referred to as ‘treeline’ throughout. Sampling was conducted in eleven heath areas north of the treeline (i.e. in subzone E or arctic) in the vicinities of the towns of Honningsvåg, Nordkapp, Mehamn, Gamvik and Berlevåg, choosing the most northerly areas available. The southern boundary of these heaths lay approximately between 70–71°N and is dominated by *Betula pubescens* Ehrh. (Refer Bandekar and Odland [26] for definition of forest limit and treeline) (Fig 1). Twelve areas were selected on heaths south of the treeline i.e. boreal and alpine heaths in the vicinity of the towns of Sørøya, Hammerfest, Gamvik, Båtsfjord and Berlevåg. The sample plots were located from 21 to 337 m a.s.l., with slopes ranging from 0–15 degrees.

Bear Island is situated halfway between Finnmark and Svalbard archipelago. The island is ca. 178 km^2^ in area. The sampling on Bear Island was conducted near the meteorological station along the north coast, which has a maritime climate [41], low average elevation and bedrock comprised of dolomite, limestone, sandstone and shale. Peat deposits are common on Bear Island. Five plots were established on relatively flat, well-drained terrain at altitudes ranging from 6 to 52 m a.s.l. Bear Island is isolated, with a steep coastline surrounded by rough seas. As we were unable to gain access, the staff of the meteorological station planted soil temperature data loggers and provided photographs of the vegetation on the island. However, neither soil sampling nor full vegetation analysis could be performed. Dwarf shrubs (especially *Salix herbacea*), vascular plants, graminoids and bryophytes could be discerned in the photographic material.

Samples from Spitsbergen were collected at Adventsdalen, ca. 10 km east of the main town of Longyearbyen. Adventsdalen branches off the inner, southeastern end of the Isfjord, the largest fjord on Spitsbergen. Adventsdalen is less prone to fog than surrounding area, and conditions are somewhat warmer than at more exposed coastal locations [41, 42]. The bedrock comprises largely of sedimentary rocks such as sandstone and shale. The dominant soil type is cryosol [40]. Nine plots were established, at elevations ranging from sea level to 482 m a.s.l., and with slope varying from 0 to 25 degrees.

Wild and domesticated grazers such as reindeer, birds and other herbivores are common throughout the study areas on Spitsbergen and Finnmark ([43] and references therein). There are no reindeer or other large herbivores on Bear Island. For conducting research on Norwegian mainland and adjoining Islands we did not require permits for sampling.

### Temperature data collection and analysis

Air temperature data from the coastal areas of Finnmark, Bear Island and Spitsbergen during the study period was obtained from 1 km^2^ resolution gridded datasets provided by Norwegian Meteorological Institute [29, 44]. From this data, air temperatures at 01:00 and 13:00 at a height of 2 m above the surface were estimated and used to calculate average daily air temperature for each site.

At the center of each quadrat, a TRIX8 temperature data-logger (LogTag recorders limited, Auckland, New Zealand) was buried 10 cm below the soil surface. Soil temperatures were recorded twice daily, at 01:00 and 13:00; the average of these two values was the daily average temperature estimate. These data were then used to derive other soil temperature variables. Definitions of the soil and air temperature variables are given in Table 1. Growth season-related temperature variables, such as start of growing season and growing season length, were calculated using a threshold value of 5°C. In order to facilitate analysis and presentation, the data series, which ran from August 2013 to August 2014 was reorganized in order to form a continuous synthetic year running from January 2014 to August 2014 followed by August 2013 to December 2013. This allowed date variables to be expressed in terms of DOY (day of year) but does not otherwise affect interpretation of the data.

**Table 1:** Overview of soil and air temperature variables, with abbreviations and measurement units used in the context of this study.

Abbr.	Definition
Avg	Average annual temperature (°C).
Max	Maximum temperature (°C).
Min	Minimum temperature (°C).
Avg(Jul)	Average July temperature (°C).
GSST	Average soil temperature during the growing season (°C).
GSAT	Average air temperature during the growing season (°C).
STHS/ATHS	Heat sum for soil (STHS) and air (ATHS) temperature is the sum of all daily average soil and air temperatures ≥ 5°C respectively, measured throughout the study period (Degree days (dd)).
STFS/ATFS	Frost sum for soil (STFS) and air (ATFS) temperature is the sum of all daily average soil and air temperatures ≤ 0°C respectively, measured throughout the study period. (Degree days (dd)).
SWI	Summer warmth index (SWI) is the sum of mean monthly temperatures greater than > 0°C [2, 22].
SGS	Start of growing season (SGS) is measured as DOY when soil temperature rose to 5°C for 5 consecutive days.
GSL	Growing season length (GSL) is measured as the number of days between SGS and the DOY when temperature (air and soil temperature) in autumn was last recorded to be 5°C.
ThD	Thaw days (ThD) is measured as number of days between snowmelt (i.e. when ground temperature was ≥ 1°C) and start of growing season (SGS).
SF	Soil frozen period (SF) is the number of days when soil temperature was ≤ 0°C (Days).

In each plot, the above variables were estimated. The variables Avg, Avg(July), and GSL were calculated for air and soil temperatures and will be followed by (A) and (S) respectively in further analysis.

### Soil sampling and analysis

Soil samples were collected at the end of the study period in August 2014. The upper 5 cm of the soil was sampled from the four corners of each vegetation plot using a 7 cm diameter steel cylinder. Soil samples from the four corners in each plot were mixed, air-dried and passed through a 2 mm sieve. Soil moisture was estimated by ASTM D 2216 (https://www.astm.org/Standards/D2216). Soil samples were analyzed for pH and plant-available Phosphorus (P), Calcium (Ca), Magnesium (Mg) and Potassium (K), by the ammonium lactate method [45] using a Perkin Elmer HGA 900 (Graphite furnace) and AIM 3000 series Flame atomic absorption spectroscope. Organic matter content was estimated by the loss-on-ignition method [46]. Bulk density (BD) was estimated using the method of Page-Dumroese and Jurgensen [47].

Two-way ANOVA was applied to determine the individual and interaction effects of latitude and altitude on each of the soil properties. Altitude was divided into two levels, low (< 200 m a.s.l.) and high (> 200 m a.s.l.) and latitude into three levels (70°N, 71°N and 78°N). Furthermore, one-way ANOVA was used to test differences in the mean values of each soil property for heaths in Finnmark north and south of the treeline and at high and low altitude. A post-hoc Tukey test was used to determine which of the groups were significantly different from one another. Analyses were performed in Minitab [48], R software [49] or Canoco 5 [50].

### Vegetation data collection and analysis

At each selected site, a 2 x 2 m quadrat was placed randomly within an area of homogeneous heath vegetation. Vascular plants were identified according to Lid and Lid [51]; the most prevalent cryptogams were identified according to Frisvoll et al. [52] and Holien and Tønsberg [53]. Abundances were estimated visually and expressed as percentage cover. Classification of vegetation plot data analysis was performed with the WinTWINS program [54] which classifies samples and species hierarchically and produces a two-way table based on pseudospecies values. Taxa were assigned to pseudospecies based on identity and cut levels (0, 5, 10, 20, 40 and 60). Taxa with less than 2 occurrences were excluded. Species occurrence and abundance (SOA) within the selected communities identified were expressed as SOA-values [55].

Canonical correspondence analysis (CCA) with interactive forward selection and Bonferroni correction was conducted to determine which of the temperature and soil properties best explained the main floristic gradients. Percentage species cover estimates were square root transformed, and all environmental variables were Log_10_ transformed and standardized for CCA analysis. Detrended correspondence analysis (DCA) analysis was run to obtain the main floristic gradients. The most important environmental variables derived from the CCA analysis were included as supplementary variables.

The data on vegetation, air temperature, soil temperature and soil properties that support the findings of this study are openly available in figshare at https://doi.org/10.6084/m9.figshare.12866261.

## Results

### Vegetation communities and associated environmental variables

Altogether, 72 plant taxa, including 56 vascular species, were recorded. Vascular plant species richness did not vary greatly with latitude; the number of species in Finnmark heaths south of treeline, north of treeline and on Adventsdalen being 29, 25 and 26 respectively.

TWINSPAN analysis divided the vegetation communities into five groups: 1. *Salix herbacea*—*Carex bigelowii* (SC); 2. *Empetrum nigrum*—*S*. *herbacea* (ES); 3. *E*. *nigrum*—*B*. *nana*—*Ptilidium ciliare* (EBP); 4. *Dryas octopetala*—*Cassiope tetragona—Poa arctica* (DCP) and 5. *Alopecurus magellanicus—Sanionia uncinata* (AS). Groups SC, ES and EBP were found in Finnmark; SC and ES were confined to high elevation plots, while EBP occurred at both low and high elevations. SC had floristic characteristics similar to snow-bed communities. Groups DCP and AS were found on Adventsdalen at low and high elevations respectively. The results of TWINSPAN analysis are shown in Table 2.

**Table 2:** Results of TWINSPAN classification.

Vegetation type	SC	ES	EBP	DCP	SA
*Arctous alpinus*			11		
*Betula nana*		17	55	7	
*Empetrum nigrum*		63	96		
*Phleum alpinum*		3	7		
*Vaccinium myrtillus*		3	7		
*Vaccinium vitis-idaea*			25		
*Pleurozium schreberi*		10	18		
*Ptilidium ciliare*		3	46		6
*Juncus trifidus*		7	6		
*Loiseleuria procumbens*		10	6		
*Cetraria ericetorum*		10	5		
*Cladonia arbuscula*		23	13		
*Ochroleuca frigida*		20	14		
*Racomitrium lanuginosum*		23	1		
*Avenella flexuosa*	6	3	1		
*Carex bigelowii*	56	23	8		
*Carex lachenalii*	17				
*Salix herbacea*	72	37	7		
*Festuca vivipara*	11	23	4	3	
*Hylocomium splendens*			2		11
*Sanionia uncinata*	33		11	13	33
*Alopecurus magellanicus*				3	33
*Cerastium arcticum*				3	11
*Cassiope tetragona*				40	
*Draba ssp*				7	6
*Dryas octopetala*		3		50	
*Luzula arctica*				20	6
*Oxyria digyna*				10	
*Poa arctica*				40	

Five communities separated by TWINSPAN were: 1. *Salix herbacea—Carex bigelowii* type (SC), 2. *Empetrum nigrum—Salix herbacea* type (ES), 3. *Empetrum nigrum—Betula nana—Ptilidium ciliare* type (EBP), 4. *Dryas octopetala—Cassiope tetragona—Poa arctica* type (DCP) and 5. *Sanionia uncinata—Alopecurus magellanicus* type (SA). Species values are given as species abundance and occurrence values (SOA) values, calculated according to Odland et al (48).

CCA analysis suggests that maximum air temperature explained most variation (17.2%, *p* = 0.002), followed by soil temperature frost sum (7.3%, *p* = 0.002), air temperature growing season length (6.5%, *p* = 0.002), soil moisture (5.4%, *p* = 0.002) and air temperature frost sum (4.3%, *p* = 0.006). Eigenvalues and explained fitted variation on axes 1, 2 and 3 were 0.79 / 44.10, 0.35 / 63.55 and 0.30 / 80.57 respectively.

**Fig 2:**
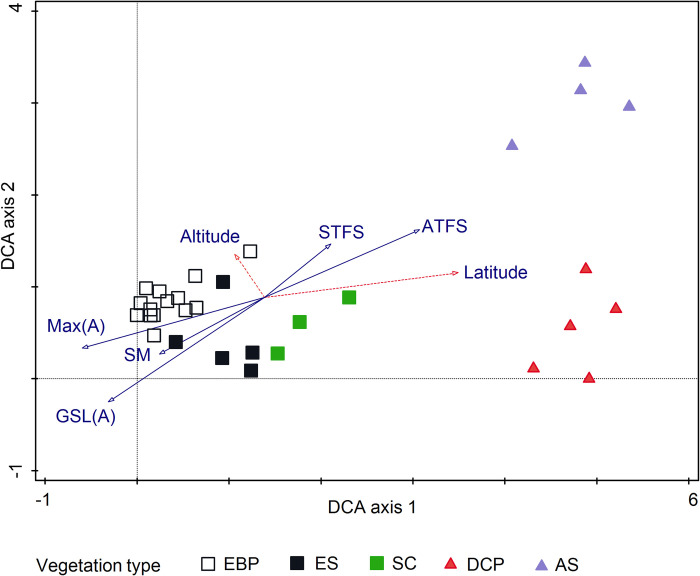
DCA diagram showing clustering of vegetation communities based on TWINSPAN analysis. The main environmental variables (temperature variables and soil properties) obtained from CCA with interactive forward selection, and altitude and latitude have been added as supplementary data. The vegetation types EBP = *E*. *nigrum–B*. *nana–P*. *ciliare*, ES = *E*. *nigrum–S*. *herbacea*, SC = *S*. *herbacea–C*. *bigelowii*, AS = *A*. *magellanicus–S*. *uncinata* and DCP = *D*. *octopetala–C*. *tetragona–P*. *arctica*. The total variation was 4.39. Max(A) = Maximum air temperature (°C), STFS = soil temperature frost sum (dd), GSL(A) = growing season length (based on air temperature) (days), SM = soil moisture (%) and ATFS = air temperature frost sum(dd).

DCA analysis was used to explore floristic gradients between the study plots (Fig 2). The vegetation clusters from TWINSPAN analysis and the temperature variables and soil properties selected through CCA were included as supplementary variables. A cumulative Eigenvalue of 1.39 was obtained with gradient lengths for axes 1, 2 and 3 as 5.45, 3.44 and 2.01 respectively. DCA axis 1 separates Finnmark and Spitsbergen; and was explained by temperature and soil properties. DCA axis 2 displays a gradient from low to high elevations. On Spitsbergen, there was a clear distinction between low-altitude (*Dryas octopetala—Cassiope tetragona—Poa arctica*) and high-altitude *(Sanionia uncinata—Alopecurus magellanicus)* communities (Fig 2, lower part of DCA axis 2). The low and high elevation heaths in north Norway were less well separated (DCA axis 2 gradient length < 1.5 SD units), indicating small floristic and temperature differences.

### Differences in temperature variables

Average air- and soil temperature data from the study plots (Table 3) show major differences in temperature variables between Finnmark and Spitsbergen, with data from Bear Island in an intermediate position, although the number of days taken to thaw the soil (Thaw Days) was lowest at Bear Island (12 ± 3.4 days). Temperature data from plots south and north of treeline in Finnmark showed minor differences (Fig 2, Table 3).

**Table 3:** Average air (A) and soil (S) temperature variables and soil properties from the different study areas.

	Finnmark		Finnmark		Bear Island	Spitsbergen	
	South of treeline		North of treeline				
Elevation	Low, *n* = 3	High, *n* = 9	Low, *n* = 6	High, *n* = 5	Low, *n* = 5	Low, *n* = 6	High, *n* = 3
Avg(S)	2.9±0.1	2.4±0.6	2.8±0.4	2.9±0.3	1.2±0.6	-1.7±0.9	-4.3±0.7
Max(S)	14.9±4.1	15.3±2.4	13.4±2.4	14.0±1.6	10.0±1.3	9.9±2.1	10.9±0.8
Min(S)	-5.2±3.4	-4.9±4.4	-5.7±3.3	-1.4±1.2	-7.0±3.4	-10.5±3.3	-15.4±3.2
STHS	1043.0±66.0	973.0±71.0	1083.0±182.0	939.7±95.0	694.0±100.0	397.0±123.0	267.0±76.0
STFS	-134.0±88.0	-230.0±221.0	-189.0±120.0	-37.0±30.0	-396.0±237.0	-1140.0±333.0	-1952.0±298.0
SF	99.0±26.0	111.0±70.0	128.0±46.0	61.0±39.0	181.0±38.0	232.0±5.0	255.0±4.0
ThD	16.0±2.0	16.0±11.0	23.0±13.0	13.0±10.0	12.0±3.0	22.0±7.0	20.0±6.0
SGS	155.0±1.0	171.0±12.0	162.0±13.0	170.0±10.0	164.0±10.0	176.0±6.0	185.0±9.0
Avg(JulS)	10.4±1.1	11.3±0.8	10.4±1.5	10.9±1.2	8.1±1.1	6.9±0.9	6.2±0.8
GSL(S)	128.0±1.0	109.0±13.0	120.0±13.0	110.0±9.0	104.0±11.0	61.0±13.0	49.0±10.0
GSST	8.5±0.4	9.2±0.4	9.0±0.7	8.8±0.4	7.0±0.7	6.5±0.6	6.0±0.4
SWI(S)	39.3±1.6	36.2±2.3	39.5±4.8	35.5±3.1	26.5±2.7	18.6±3.0	13.6±1.5
Avg(A)	3.3±0.7	2.0±0.9	2.6±0.2	2.9±0.4	0.2±0.1	-2.3±0.1	-4.7±0.2
Max(A)	19.9±0.3	18.2±1.2	17.6±1.0	17.2±0.9	10.2±0.1	10.6±0.1	8.2±0.2
Min(A)	-9.8±1.7	-12.1±2.2	-10.3±0.4	-10.0±0.3	-11.4±0.1	-18.0±0.1	-20.4±0.2
ATHS	1348.0±97.0	1089.0±156.0	1164.0±29.0	1121.0±104.0	449.0±15.0	489.0±17.0	191.0±31.0
ATFS	-362.0±112.0	-551.0±196.0	-412.0±51.0	-291.0±24.0	-600.0±16.0	-1493.0±22.0	-2055.0±44.0
Avg(JulA)	12.8±1.1	11.0±1.3	10.6±0.5	10.2±0.7	6.2±0.1	7.1±0.1	4.7±0.2
GSL(A)	141.0±7.0	129.0±6.0	129.0±0.0	130.0±2.0	85.0±0.0	83.0±1.0	46.0±3.0
GSAT	9.9±0.3	8.8±0.8	9.1±0.2	8.8±0.6	6.2±0.1	6.5±0.1	4.7±0.0
SWI(A)	48.0±3.6	39.0±5.5	41.7±0.9	41.0±3.7	21.5±0.4	21.4±0.4	11.9±0.7
pH	4.3±0.1	4.6±0.5	4.6±0.4	5.2±0.3		5.5±0.4	5.5±0.3
P	7.2±0.5	3.3±2.1	6.0±3.2	2.7±0.5		3.5±1.5	2.0±0.1
Ca	142.7±30.2	114.1±128.7	179.1±130.1	146.0±73.9		244.0±154.4	133.7±25.5
Mg	133.2±14.4	51.4±52.3	108.5±66.9	84.7±38.4		43.1±14.9	36.8±5.3
K	46.2±3.2	20.8±16.9	37.3±18.7	18.9±5.4		11.6±3.7	12.4±2.6
OM	80.5±8.5	27.1±26.2	56.6±33.8	28.4±12.4		13.0±5.0	7.0±2.6
SM	67.4±1.3	38.6±14.3	53.6±19.9	44.2±10.8		34.3±7.1	15.5±5.1
BD	0.2±0.0	0.7±0.3	0.5±0.4	0.6±0.2		0.7±0.2	1.0±0.0

The averages are reported for both high and low elevation plots in separate columns with standard deviation values (±SD) and *n* = sample size. Abbreviations and terminologies used here for temperature variables along with their units of measurement are explained in Table 1. Soil properties include; pH, P = phosphorus (mg/100g), Ca = calcium (mg/100g), Mg = magnesium (mg/100g), K = potassium (mg/100g), OM = organic matter (%), SM = soil moisture (%) and BD = bulk density (g/cm^3^).

The effect of altitude on temperature variables differed between Finnmark and Adventsdalen. Regression analysis (S2 Table) showed correlations only for SGS, GSL (S) and GSAT in Finnmark, while on Adventsdalen, strong correlations were seen for Avg(S), STFS, SF, ATHS, ATFS, GSAT and GSL (S2 Table).

Growing season length was two weeks longer at low altitudes compared to high altitudes in Finnmark, growing season length at high altitude was more or less equal to Bear Island, and much longer than on Spitsbergen (Table 3). Heath plots- south and north of treeline did not differ significantly. The same applied to growing season temperature, growing season length, heat sum and average July temperature.

The influence of altitude on temperature variables was stronger on Spitsbergen than in Finnmark, more temperature variables were significantly correlated with altitude and the correlations were stronger for Spitsbergen than for Finnmark (S2 Table).

### Variation in soil properties

Soil property data is shown in Table 3. Alkalinity increased with latitude. Plant available soil nutrients (with exception of Ca and BD), OM and SM were higher in Finnmark as compared to Spitsbergen, while BD was highest in Spitsbergen. A two—way ANOVA showed significant correlations between latitude and pH, K, SM, and OM, and also between altitude and P, Mg, K, SM, BD and OM. Median soil property values were similar for plots north and south of the treeline in Finnmark at both high and low altitudes (Mann-Whitney test results given in S3 Table).

## Discussion and conclusion

### Latitudinal and altitudinal differences in vegetation, temperature variables and soil properties

Vegetation variation of heaths in North Norway, Bear Island and Spitsbergen conducted in our study comply well with previously described literature [2, 11, 13, 20, 27, 56–58]. Studies have been performed along gradients of latitude, altitude, oceanity, air temperature, precipitation, effect of the North Atlantic current (west to east gradient) and ground conditions such as permafrost and bedrock [23, 27, 59–62].

The heath vegetation plots on Spitsbergen were separated into two groups: DCP and AS. DCP was prevalent at low altitude areas and the floristic composition, which included *Dryas octopetala* and *Cassiope tetragona*, together with some *Poa arctica* and *Betula nana*, is indicative of exposed and dry conditions. Such species were lacking in AS, which was prevalent at higher altitudes and was dominated by *Alopecurus magellanicus*. DCP heaths had relatively well-drained, dry minerogenic soils which thawed quickly, which suggests that soil frost plays a relatively minor role in this community. Similar rapid thawing has been found in *Dryas* heaths in the alpine zone in south Norway [63]. DCP and AS resemble, respectively, the *Cassiope tetragona—Hylocomium* (CtHC) and *Hylocomium—Tomenthypnum—Saniona* (HTSaC) communities previously described from Spitsbergen [16, 43].

The study plots from Finnmark were separated into three groups, two were mainly situated at high altitude (SC and ES), and one in lowland coastal areas (EPB). These communities strongly resemble vegetation communities previously described from Finnmark. Both the ES and the EPB types show strong similarities with the Hemiarctic empetrum—lichen type described by Haapasaari [11]. The SC type corresponds to moderate snowbed vegetation as described by Gjærevoll [64] as *Carex bigelowii—Carex lachenalii—Saniona uncinata* associations.

The three communities in Finnmark were floristically strongly related and separated by less than 2 SD units on the DCA axis 2 (Fig 2). The small differences found between vegetation communities at low and high elevation are reflected in similarities in growing season length and growing season temperature (Table 3).

In Finnmark, the soils were acidic, potassium-rich, and showed a relatively thick humus layer, and the plant communities were dominated by acidophiles. The heath plots on Spitsbergen were located on minerogenic soils with low content of organic matter, low soil moisture, and high bulk density compared with the Finnmark soils (Table 3). As a result, permafrost melts relatively early and substrate does not become soggy during summer. The soils were Ca-rich, and had a high pH, and the plant communities were dominated by calciphiles, which is in agreement with previous findings [31, 58]. Boreal soils are characteristically acid podzols [40]. In the present case, two other factors need to be taken into account: precipitation (www.eklima.no; 27), which is 3-fold higher (650 mm yr^-1^) in Finnmark than on Spitsbergen (210 mm yr^-1^), and the bedrock, which is predominantly base-rich on Spitsbergen [58] and acidic in Finnmark. No significant differences in soil chemistry were found between heaths south and north of the treeline, in Finnmark.

The average annual air temperatures during the study period at Finnmark, Bear Island and Spitsbergen were 1.0°C, 2.6°C, 3.8°C higher, and the average July air temperatures were 0.5°C, 1.8°C and 0.7°C higher than the 1961–1990 normals respectively [65].

Regression analyses for the Finnmark plots show that start of growing season, growing season length based on soil temperatures and growing season air temperature did not vary significantly with altitude. This is surprising since the plots are separated by an elevation of approximately 300 m. This may be explained by the close vicinity to the sea and strong wind. According to Karlsen et al. [62], the earliest onset of the growing season in Finnmark was found in the narrow strip of lowland between the mountains and the sea along the coast of northern Norway. The onset followed a clear gradient from lowland to mountain corresponding to the decreasing temperature gradient. The length of the growing season is was 130 days in coastal areas and 100 days at high levels.

**Fig 3:**
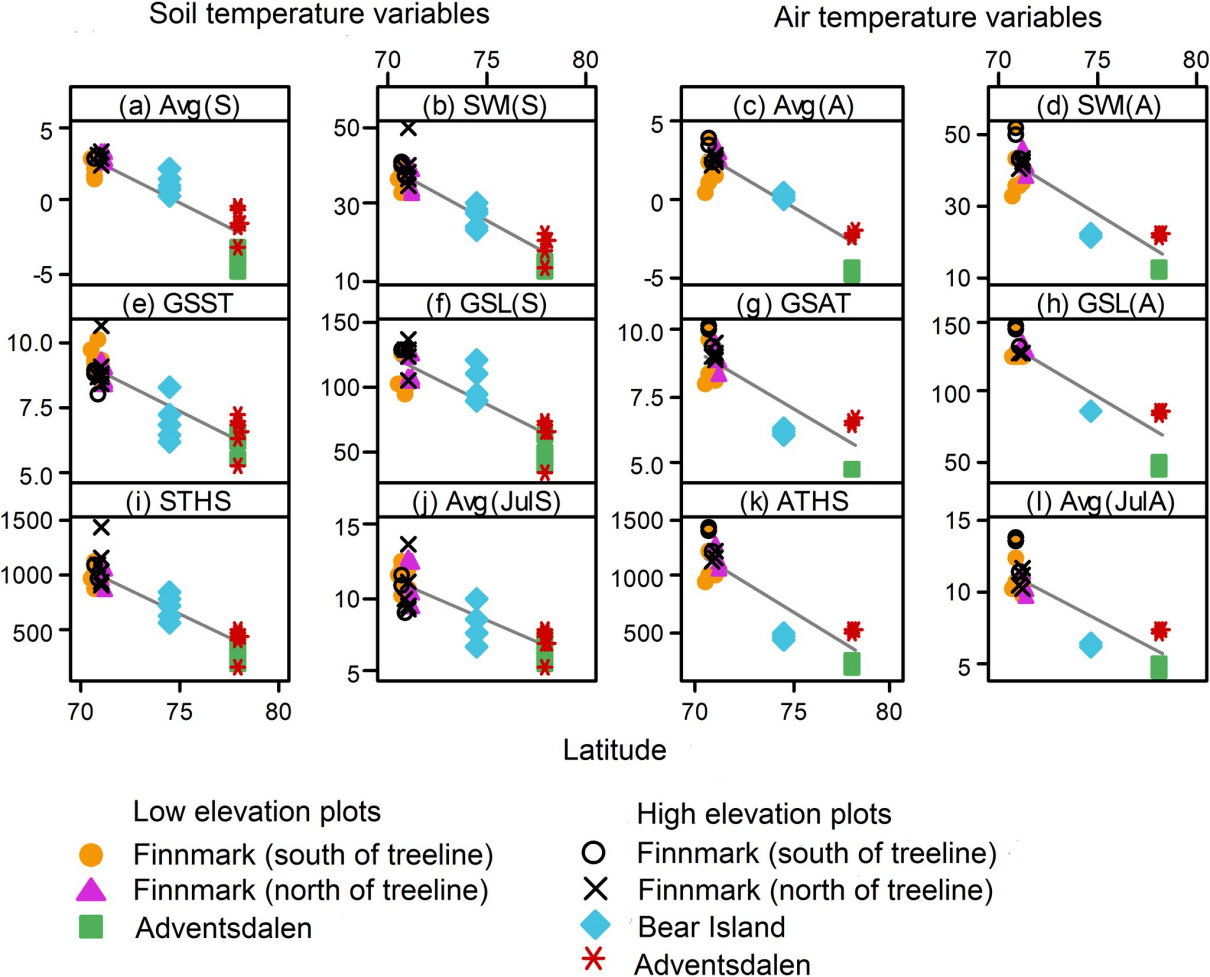
Trends of lowland soil temperature variables from northernmost Norway, via Bear Island to Spitsbergen. Abbreviations are according to Table 1. Regression equations are given in S1 Table.

On Spitsbergen the soil temperature (Avg(S)) declined significantly by 0.6°C 100 m^-1^ (S2 Table). Increasing latitude was associated with strong cooling trends as previously shown by Bliss [3]; Chernov & Matveyeva [4]; Harper et al. [6]. There were major differences in temperature variables between Finnmark and Spitsbergen and with intermediate values at Bear Island showing intermediate values (Fig 3, Table 3). Most significant were the increase of frost-related variables towards the north. Air temperature heat sums for Finnmark and Bear Island were similar to those previously reported [30, 41].

### Do coastal heaths of northernmost Norway belong to arctic?

Mean annual air temperatures in Finnmark was rarely lower than 2.5°C. Even allowing for the anomalously high temperature during the study period (+ 0.5 to + 1.0°C), these places in Finnmark lie outside of the -1°C isotherm which is considered the southern limit of discontinuous permafrost and still further outside the -8°C isotherm that characterizes the onset of continuous permafrost and true arctic conditions. Both the air and the soil temperature measurements indicate that permafrost should not occur in northernmost Norway. This supports the study of [66] who stated that there were practically no mineral soils with seasonal freezing layer reaching the permafrost table in Fennoscandia. In Finnmark, only sporadic permafrost zones, restricted to scattered palsa mires, have been reported [67]. At bedrock depths of more than 5 m, temperatures consistent with permafrost conditions are encountered, considered to be a relic of the last ice age [68–71]. Otherwise in Scandinavia, permafrost has only been detected in boreholes on high mountains [72]. This indicates that arctic permafrost conditions are not found in Finnmark.

On Spitsbergen, on the other hand, annual air and soil temperatures were mostly lower than -2°C. The upper layers of soil in heath communities studied (upper 10 cm of the soil) melted during summer both on Spitsbergen and northernmost Norway. On Spitsbergen, average annual soil temperature was—4.3 ± 0.9°C at high elevation (average 450 m) and—1.7 ± 1.0°C at low elevation (average 20 m). Average July soil temperature was 6.2 ± 0.9°C at high elevation and 6.9 ± 1.0°C at low elevation.

In temperature terms, the arctic has been defined as having mean annual temperature < 0°C, mean July temperature < 10°C, and growing season < 60 days [73, 74], as against > 80 days for the boreal [74–76]. Summer warmth index for arctic subzone E is given as 26.5–29.5. None of these conditions are met in our data for Finnmark, wherein the average annual air- and soil temperature was 1.1–4.1°C (air) and 1.8–3.2°C (soil); the mean July temperature was 9.5–13.9°C (air) and 8.9–12.1°C (soil); the growing season was 125–148 days (air) and 96–133 days (soil) and the summer warmth index was 33.5–51.6 (air) and 33.9–44.3 (soil).

It has previously been shown that the distribution limit of forests in Finnmark might not be limited by air- and soil temperatures, which were higher than those found in woodlands near the alpine treeline in southern Norway, but rather owing to lack of suitable growth areas [26]. Similarly, most of the Finnmark heath plots, both north and south of the treeline had climatic conditions within the range encountered in forests at high elevation [77, 78]. Therefore, the northern limit for tree growth in Finnmark is probably determined by other factors such as wind exposure, steep topography and extensive areas of bare rock [25, 79], rather than by temperature. Thus, it is not an arctic treeline in the normal sense of the term.

We found no significant differences in air and soil temperatures, soil properties or vegetation in heaths north and south of the treeline. This indicates that there is not any reason for separating these areas into different biomes. Previous studies have excluded heaths north of the treeline in Norway and Kola Peninsula from the arctic zone due to dominance of large number of southern boreal species [8, 12, 13, 80]. Ahti et al. [8], Haapasaari [11], Eurola [31] and Böcher [79] maintained that nowhere in Fennoscandia is there truly regional arctic vegetation at sea level, and that the oceanic sea-level heaths largely belong to the mainly wooded northern boreal zone.

Whether the treeless coastal heaths of northernmost Norway are arctic or not have been discussed for more than seventy years [8, 20, 31, 81, 82]. Our findings agree with previous studies that conclude that northern Finnmark is not a part of the arctic biome. Temperature variables are higher than reported for arctic areas and too high to allow permafrost. There is no significant difference in heath vegetation north and south of the treeline. We therefore agree with Ahti et al. [8] that coastal areas of northern Finnmark should be considered oceanic sections of the northern boreal biome.

## Conclusion

Our results suggest that the boreal biome extends all the way to the north coast of mainland Norway; and previously used division of heaths in Finnmark into boreal, alpine and arctic biomes is not justified. Both soil conditions, air- and soil temperatures, length of growing season and coastal vegetation in Finnmark are in line with what is typical for the boreal biome.

## Supporting information

S1 Table Results of regression analysis with temperature variables as response and latitude as predictor. Separate analyses have been performed for data from low and high plots because there were no high elevation plots at Bear Island. All the abbreviations with their units of measurement are explained in Table 1.
(PDF)

S2 Table Results from regression analysis with temperature variables as response and altitude as predictor. Separate analysis are conducted for Finnmark and Adventsdalen (Only results with significant p values (< 0.05) have been given in the table). All the abbreviations with their units of measurement are explained in Table 1.
(PDF)

S3 Table Results of Mann-Whitney test for comparing the means of south- and north of treeline plots in Finnmark. The results of the analysis were *p* > 0.05 for all variables indicating that there is no a statistically significant difference in medians between the two heath zones. *W* = Wilcoxon test statistic.
(PDF)
