# Supplementary material for: Bioclimatic gradients and soil property trends from northernmost mainland Norway to the Svalbard archipelago. Does the arctic biome extend into mainland Norway?
Source: PLoS One. 2020 Sep 17;15(9):e0239183. doi: 10.1371/journal.pone.0239183 (PMC7498165; doi:10.1371/journal.pone.0239183)
Supplement: S1 Table — Separate analyses have been performed for data from low and high plots because there were no high elevation plots at Bear Island. All the abbreviations with their units of measurement are explained in Table 1. (PDF) [file pone.0239183.s001.pdf]

S1 Table. Results of regression analysis with temperature variables as response and latitude as predictor. Separate analyses have been performed for data from low and high plots because there were no high elevation plots at Bear Island. All the abbreviations with their units of measurement are explained in Table 1.

Regression equation (high elevation plots) *n* = 17	*rs*	*p*	Regression equation (low elevation plots) *n* = 20	*rs*	*p*
Avg(S) = 68.71 - 0.93*Latitude	0.93	0.000	Avg(S) = 46.44 - 0.6129*Latitude	0.88	0.000
Max(S) = 53.46 - 0.54*Latitude	0.32	0.010	Max(S) = 55.23 - 0.5881*Latitude	0.29	0.008
Min(S) = 105.8 - 1.54*Latitude	0.50	0.001	Min(S) = 43.05 - 0.6818*Latitude	0.24	0.016
STHS = 7748 - 95.74*Latitude	0.92	0.000	STHS = 7658 - 93.03*Latitude	0.81	0.000
STFS = 16969 - 241.70* Latitude	0.87	0.000	STFS = 9120 - 130.2*Latitude	0.70	0.000
SF = - 1399 + 21.08*Latitude	0.43	0.002	SF = - 1006 + 15.86*Latitude	0.66	0.000
Avg(JulS) = 59.27 - 0.67*Latitude	0.79	0.000	Avg(JulS) = 45.41 - 0.49*Latitude	0.59	0.000
ThD = - 23.64 + 0.54*Latitude	0.00	0.556	ThD = 7.17 + 0.15*Latitude	0.00	0.827
SGS = 34.20 + 1.92*Latitude	0.14	0.08	SGS = 3.70 + 2.18*Latitude	0.31	0.006
GSL(S) = 694.4 - 8.25*Latitude	0.79	0.000	GSL(S) = 716.6 - 8.34*Latitude	0.80	0.000
GSST = 38.77 - 0.41*Latitude	0.87	0.000	GSST = 32.01 - 0.32*Latitude	0.62	0.000
Avg(A) = 69.86 - 0.95*Latitude	0.89	0.000	Avg(A) = 53.23 - 0.71*Latitude	0.98	0.000
Max(A) = 113.4 - 1.34*Latitude	0.95	0.000	Max(A) = 98.12 - 1.13*Latitude	0.74	0.000
Min(A) = 74.80 - 1.21*Latitude	0.73	0.000	Min(A) = 65.25 - 1.05*Latitude	0.85	0.000
ATHS = 9993 - 125.50*Latitude	0.88	0.000	ATHS = 8754 - 107.40*Latitude	0.74	0.000
ATFS = 14819 - 215.60*Latitude	0.88	0.000	ATFS = 10081 - 146.70*Latitude	0.89	0.000
GSAT = 48.99 - 0.56*Latitude	0.87	0.000	GSAT = 38.34 - 0.41*Latitude	0.71	0.000
GSL(A) = 943.4 - 11.48*Latitude	0.97	0.000	GSL(A) = 637.7 - 7.18*Latitude	0.79	0.000
Avg(JulA) = 70.17 - 0.83*Latitude	0.83	0.000	Avg(JulA) = 55.01 - 0.62*Latitude	0.59	0.000
