# Supplementary material for: Bioclimatic gradients and soil property trends from northernmost mainland Norway to the Svalbard archipelago. Does the arctic biome extend into mainland Norway?
Source: PLoS One. 2020 Sep 17;15(9):e0239183. doi: 10.1371/journal.pone.0239183 (PMC7498165; doi:10.1371/journal.pone.0239183)
Supplement: S2 Table — Separate analysis are conducted for Finnmark and Adventsdalen (Only results with significant p values (< 0.05) have been given in the table). All the abbreviations with their units of measurement are explained in Table 1. (PDF) [file pone.0239183.s002.pdf]

S2 Table. Results from regression analysis with temperature variables as response and as predictor. Separate analysis are conducted for Finnmark and Adventsdalen (Only results with significant p values (< 0.05) have been given in the table)**.** All the abbreviations with their units of measurement are explained in Table 1

Regression equation	*rs*	*p*
Finnmark (*n* = 23)		
SGS = 157.6 + 0.04*Altitude	0.14	0.000
GSL(S) = 124.8 - 0.05*Altitude	0.19	0.021
GSAT = 9.474 - 0.002*Altitude	0.16	0.035
Adventsdalen (*n* = 9)		
Avg(S) = - 1.541 - 0.006*Altitude	0.62	0.007
STFS = - 1101 - 1.94*Altitude	0.54	0.014
SF = 230.7 + 0.05*Altitude	0.84	0.000
ATHS = 502.9 - 0.71*Altitude	0.99	0.000
ATFS = - 1467 - 1.33*Altitude	1.0	0.000
GSAT = 6.582 - 0.004*Altitude	0.99	0.000
GSL(A) = 85.07 - 0.08*Altitude	0.99	0.000
