# Supplementary material for: Bioclimatic gradients and soil property trends from northernmost mainland Norway to the Svalbard archipelago. Does the arctic biome extend into mainland Norway?
Source: PLoS One. 2020 Sep 17;15(9):e0239183. doi: 10.1371/journal.pone.0239183 (PMC7498165; doi:10.1371/journal.pone.0239183)
Supplement: S3 Table — The results of the analysis were p > 0.05 for all variables indicating that there is no a statistically significant difference in medians between the two heath zones. W = Wilcoxon test statistic. (PDF) [file pone.0239183.s003.pdf]

S3 Table. Results of Mann-Whitney test for comparing the means of south- and north of treeline plots in Finnmark. The results of the analysis were $p > 0.05$ for all variables indicating that there is no a statistically significant difference in medians between the two heath zones. *W*= Wilcoxon test statistic.

Variables	*W*	*p*
Avg(S)	145.5	0.951
Max(S)	138.5	0.758
Min(S)	148.5	0.805
STHS	143.5	1.000
STFS	147.5	0.853
SF	141.5	0.902
Avg(JulS)	138.5	0.758
ThD	142.5	0.951
SGS	141.5	0.901
GSL(S)	147	0.876
GSST	138.5	0.758
Avg(A)	139.5	0.804
Max(A)	149.5	0.755
Min(A)	138.5	0.756
ATHS	146.5	0.901
ATFS	138.5	0.756
GSAT	146.5	0.901
GSL(A)	147.5	0.840
Avg(JulA)	148.5	0.804

SWI(S)	143.5	1.000
SWI(A)	146.5	0.901
pH	139.5	0.804
P	146.5	0.901
Ca	143.5	1.000
Mg	144.5	1.000
K	147.5	0.853
SM	147.5	0.853
BD	138.5	0.758
OM	147.5	0.853
